# The Ionization of Polymeric Materials Accelerates Protein Deposition on Hydrogel Contact Lens Material

**DOI:** 10.3390/ma16052119

**Published:** 2023-03-06

**Authors:** Jihye Ahn, Moonsung Choi

**Affiliations:** 1 Department of Optometry, College of Energy and Biotechnology, Seoul National University of Science and Technology, Seoul 01811, Republic of Korea; 2 Convergence Institute of Biomedical Engineering and Biomaterials, Seoul National University of Science and Technology, Seoul 01811, Republic of Korea

**Keywords:** polymer additives, contact lens, surface characteristics, protein deposition

## Abstract

Contact lens materials include polymers that are ionized in the ocular pH condition and are susceptible to protein deposition due to their surface characteristics. Herein, we investigated the effect of the electrostatic state of the contact lens material and protein on protein deposition level using hen egg white lysozyme (HEWL) and bovine serum albumin (BSA) as model proteins and etafilcon A and hilafilcon B as model contact lens materials. Only HEWL deposition on etafilcon A showed a statistically significant pH-dependency (*p* < 0.05); protein deposition increased with pH. HEWL showed a positive zeta potential at acidic pH, while BSA showed a negative zeta potential at basic pH. Only etafilcon A showed a statistically significant pH-dependent point of zero charge (PZC) (*p* < 0.05), implying that its surface charge became more negative under basic conditions. This pH-dependency of etafilcon A is attributed to the pH-responsive degree of ionization of its constituent methacrylic acid (MAA). The presence of MAA and its degree of ionization could accelerate protein deposition; more HEWL deposited as pH increased despite the weak positive surface charge of HEWL. The highly negatively charged etafilcon A surface attracted HEWL, even overwhelming weak positive charge of HEWL, increasing the deposition with pH.

## 1. Introduction

Contact lenses are vision correction tools directly inserted into the eye that float on the ocular surface. Materials for contact lenses include charged groups of ionic polymers that are ionized in the ocular pH condition, such as methacrylic acid (MAA) [1,2]. These ionic contact lens materials are susceptible to protein deposition due to their surface characteristics.

Protein deposition on contact lens materials has been extensively studied for decades [3,4,5,6,7,8,9,10,11,12,13,14]. It has been shown that there are a great many factors that affect the level of protein deposition on contact lenses, including water content, surface roughness, pore size, and charge [1,4,5,6,7,8,12,13]. For example, the remarkably high level of hen egg white lysozyme (HEWL), a positively charged protein, deposition on etafilcon A, a negatively charged contact lens material, has been attributed to the electrostatic attraction between them [5,7,8,12].

The pH of the ocular surface governs the electrostatic properties of proteins and contact lens materials due to the electrostatic properties of amino acids and polymeric additives. Both proteins and contact lens materials have a specific pH at which their surface charges become neutral; for proteins, it is called the isoelectric point (pI), while for contact lens materials, it is called the point of zero charge (PZC) [15,16,17]. For example, when the pH is above their pI and PZC, the protein and contact lens materials have a net negative surface charge and vice versa. The pI and PZC are determined by the properties of the amino acids that compose the proteins and the type of polymers and additives composing the contact lens materials. For example, etafilcon A contains pHEMA, co-polymerized with methacrylic acid (MAA), as a backbone and PVP (polyvinylpyrrolidone) as an additive [18]. MAA is a organic compound with a pK_a_ of 4.65, which has a carboxyl group as a functional group when polymerized [19]. The presence of MAA in etafilcon A gives it a pH-responsive negative surface charge at physiological conditions [20].

In this study, we investigated the protein deposition on contact lens materials in a series of pH, that are found in the ocular surface from 6.2 to 8.2, to evaluate the effect of their electrostatic state on the protein deposition level [21,22]. We tried to identify the electrostatic state which governs the protein deposition on contact lens materials in a range of possible ocular pH ranges. Two representative tear proteins that have opposite surface charges at physiological pH, HEWL and bovine serum albumin (BSA) were used; they have pIs of 10.7 and 4.5, respectively [23]. Two ionic and non-ionic hydrogel contact lens materials, etafilcon A and hilafilcon B, were used to assess the effect of the surface charge on protein deposition: etafilcon A contains methacrylic acid (MAA), which is a pH-responsive ionic monomer, while hilafilcon B contains N-Vinyl-2-pyrrolidone (NVP), which is a non-ionic monomer [20].

## 2. Materials and Methods

### 2.1. Materials

The 1-Day ACUVUE MOIST (Johnson & Johnson Inc., Guelph, ON, Canada) and SofLens daily disposable (Bausch + Lomb Inc., Laval, QC, Canada) were used (Table 1). All contact lenses were incubated in each buffer solution for 48 h to reach an equilibrium state before every experiment. The PBS buffers with different pH values from 6.2 to 8.2 were prepared using sodium chloride, potassium chloride, sodium phosphate dibasic, and potassium phosphate monobasic (Sigma-Aldrich, Waltham, MA, USA). In addition, HEWL, bovine serum albumin (BSA), potassium nitrate, nitric acid, potassium hydroxide, and hydrochloric acid were used; all reagents were purchased from Sigma-Aldrich.

### 2.2. Measurement of Protein Deposition on Contact Lens

Solutions of HEWL at 1.9 mg/mL and BSA at 1 mg/mL were prepared in PBS (137 mM NaCl, 2.7 mM KCl) with pHs ranging from 6.2 to 8.2. The concentrations of HEWL and BSA in their respective solutions were confirmed by UV-vis spectrometry. Each contact lens sample was immediately added to 1 mL of each protein solution after being incubated in each PBS solution, capped, and shaken for 16 h at 35 °C. After 16 h of protein absorption, the concentrations of supernatant protein solution were measured by absorbance at 280 nm using a UV-vis spectrometer (LAMBDA 465, PerkinElmer, Inc., Waltham, MA, USA). The amount of absorbed proteins at each pH condition was calculated by subtracting the final concentration from the initial concentration using the following equation (Equation (1)) [24,25,26]. Experiments were performed in triplicate.

(1) $$m_{deposited}=m_{initial}-m_{final}-m_{plate}$$

where $m_{initial}$ is the initial content of the protein solution, $m_{final}$ is the final content of the protein solution after incubating the contact lens, $m_{plate}$ is the content of protein that the incubation plate absorbs. The deposited proteins ($m_{deposited}$) are calculated by substrating $m_{final}$ and $m_{plate}$ from $m_{initial}$.

### 2.3. Measurement of Zeta Potential of Lysozyme and BSA

The zeta potential of HEWL and BSA was measured by a dynamic laser light scattering method in PBS solutions with a pH ranging from 6.2 to 8.2 using a zeta potentiometer (ELSZneo, Otsuka Electronics, Osaka, Japan). Experiments were performed in triplicate.

### 2.4. Measurement of PZC of Contact Lens

The contact lens samples were equilibrated in PBS solutions with pHs ranging from 6.2 to 8.2 for 48 h. The pH dependence of the PZC of the contact lenses was measured using 0.1 N KNO_3_ as a background electrolyte. The initial pH solutions ranged from 4.0 to 9.0, and each pH value was adjusted using either 0.1 N nitric acid or potassium hydroxide. The contact lens samples were added to each conical tube, capped, and shaken for 48 h to reach an equilibrium at room temperature. The final pH values ($pH_{final}$) of the supernatant liquids were then measured. The change in pH, ΔpH ($\Delta pH=pH_{fianl}-pH_{initial}$) was plotted against $pH_{initial}$ and the point of intersection of the curve where ΔpH=0 is the $pH_{PZC}$ value of a sample contact lens [16,17]. Experiments were performed in triplicate.

### 2.5. Measurement of Hydrooxyl Group Content of Contact Lens

The content of hydroxyl groups per gram of etafilcon A and hilafilcon B in a series of pH was determined by the titration method. Each contact lens was added to 25 mL of hydrochloric acid (0.001 M) and incubated overnight at room temperature. The titration was carried out in triplicate. The hydroxyl group content per gram of etafilcon A and hilafilcon B was calculated using the following equation (Equation (2)) [27,28].

(2) $$C=\frac{C_{i}-C_{f}}{m}V$$

where $C_{i}\left(\mathrm{mol}\cdot L^{-1}\right)$, $C_{f}\left(\mathrm{mol}\cdot L^{-1}\right)$, and $V\left(\mathrm{mL}\right)$ are the initial and final concentrations of the hydrochloric acid solution, respectively, $V\left(\mathrm{mL}\right)$ is its volume, and $m\left(mg\right)$ is the mass of etafilcon A or hilafilcon B in a series of pH. Experiments were performed in triplicate.

### 2.6. Statistical Analysis

IBM SPSS Statistics (for Windows, Version 29.0.0., SPSS Inc., Armonk, NY, USA) was used to perform statistical procedures. The Kruskal–Wallis test was used to analyze the data from the non-normal population. The level of significance was set at *p* < 0.05.

## 3. Results and Discussion

### 3.1. Protein Deposition on Contact Lens

**Figure 1:**
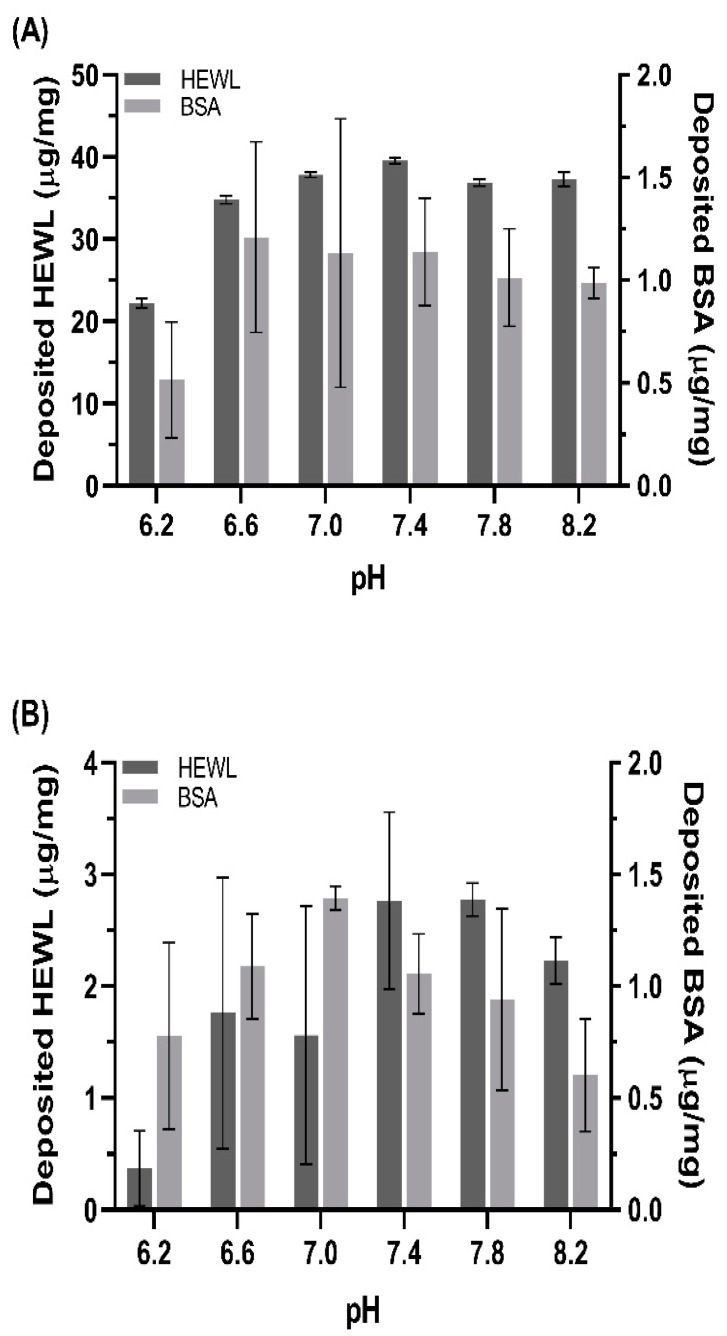
The amount of deposited hen egg white lysozyme (HEWL, 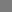) and bovine serum albumin (BSA, 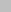) on (**A**) etafilcon A and (**B**) hilafilcon B in a range of pH.

The etafilcon A and hilafilcon B contact lenses were incubated with HEWL and BSA, respectively. The results of protein deposition on etafilcon A and hilafilcon B are shown in Figure 1. The level of HEWL deposition on etafilcon A and hilafilcon B was about 20- and 2-fold greater than that of BSA, respectively. This large protein-dependent deposition has been widely reported [5,7,12]. This is attributed to the small size of HEWL that easily penetrates through the hydrogel [5,12].

Only the level of HEWL deposition on etafilcon A was affected by the pH to a statistically significant degree (Table 2). This phenomenon is expected to be due to electrostatic interaction since etafilcon A and HEWL are expected to have negative and positive surface charges, respectively. The lower level of negatively charged BSA deposition on etafilcon A than HEWL supports this hypothesis. In recent reviews that described the chemical composition of soft materials and their properties, the preferable type of protein to deposit on the materials depending on the type of monomers is summarized [23,29]. According to those reviews, MAA gives a material negative surface charge, therefore attracting positively charged proteins such as lysozyme.

The non-ionic contact lens material, hilafilcon B, showed a lower level of protein deposition than etafilcon A. The higher level of deposition of HEWL than BSA is expected to be attributed to its small size, which is not an electrostatic interaction [12]. Therefore, we investigated the respective surface charges of the proteins and contact lenses to examine the driving force behind protein deposition on contact lenses.

### 3.2. Zeta Potential of HEWL and BSA

**Figure 2:**
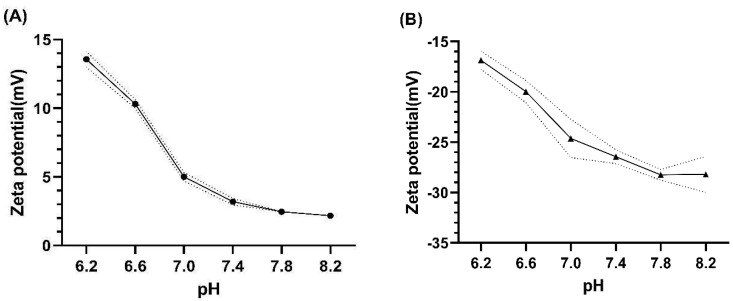
Zeta potential values of (**A**) HEWL and (**B**) BSA in a range of pH.

The zeta potentials of HEWL and BSA are shown in Figure 2. The zeta potential of HEWL showed a stronger positive potential at acidic conditions and became weak at basic conditions. In contrast, the zeta potential of BSA showed a stronger negative potential at basic conditions and weaker negative potential at acidic conditions. The HEWL and BSA have pIs of 10.7 and 4.5, respectively [30,31,32]. Therefore, the net surface charge of HEWL should decrease toward zero with increasing pH, while that of BSA should become more negative under the same conditions.

Regarding the result of HEWL deposition on etafilcon A, the weak positive charge of HEWL at basic conditions seems contradictory, as it implies that the charge of HEWL has little effect on its deposition on etafilcon A.

### 3.3. Point of Zero Charge of Contact Lens

**Figure 3:**
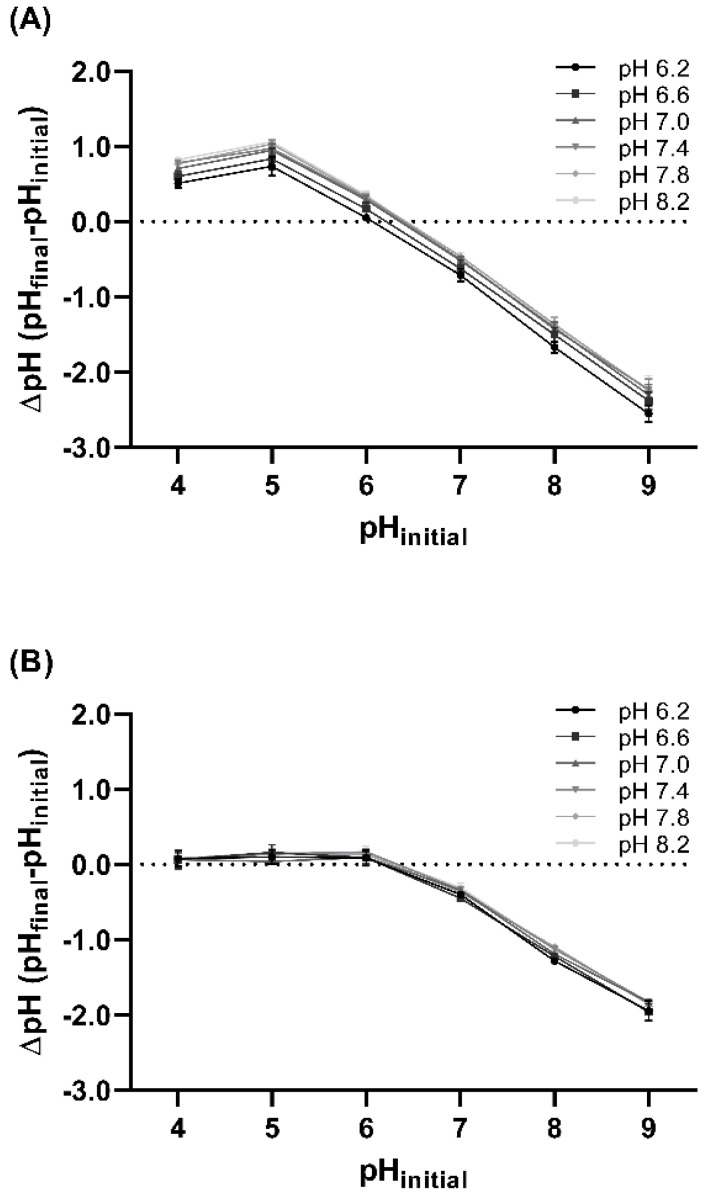

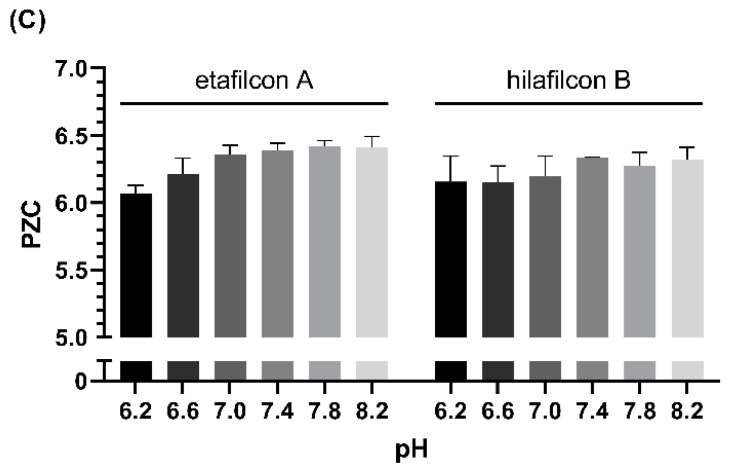
The ΔpH of (**A**) etafilcon A and (**B**) hilafilcon B and (**C**) the PZC of etafilcon A and hilafilcon B equilibrated in PBS solutions (pH 6.2–8.2).

The point of zero charge (PZC) of etafilcon A and hilafilcon B was measured to investigate their surface charge at a range of pHs (Figure 3). The PZC of etafilcon A and hilafilcon B ranged from 6.07 to 6.42 and 6.13 to 6.32, respectively. All the PZCs had lower values than each pH condition investigated, which implies that etafilcon A and hilafilcon B have negative surface charges. Furthermore, as the pH increased, the difference between PZC and pH also increased, which implies that the net negative surface charge of etafilcon A is expected to increase with pH.

The etafilcon A showed a statistically significant pH-dependent PZC trend (Table 3), showing a relatively constant value above 7.0. This pH dependence of PZC in etafilcon A is believed to be derived from the MAA. This pH-sensitive additive has a pK_a_ of 4.65 and is fully ionized at physiological pH [15,20]. The degree of ionization of MAA depends on pH and largely affects the surface charge and PZC of etafilcon A; the MAA became fully ionized at physiological pH, reaching the maximum surface charge of etafilcon A where the PZC became relatively constant.

The PZC of hilafilcon B was little affected by the pH. This would be derived from the presence and property of N-Vinyl-2-pyrrolidone (NVP), the temperature-sensitive additive [20]. As all the experiments were performed at room temperature, the degree of ionization of NVP is expected to be rarely changed, which resulted in the relatively constant PZC in the series of pH ranges.

Regarding the surface charge of HEWL and etafilcon A, the level of HEWL deposition was increased with pH despite the weaker positive surface charge of HEWL. This implies that the more negative surface charge of etafilcon A overwhelmed the weaker positive charge of HEWL at basic conditions. Therefore, the high negativity of the etafilcon A surface further attracted weakly positive charged HEWL, resulting in an increased deposition at basic pH conditions. This is supported by the fact that HEWL and hilafilcon B were not affected by pH; the weaker negative surface charge of hilafilcon B rarely attracted positive HEWL, resulting in a relatively constant level of HEWL deposition in the tested pH range. In a previous study, Kurokawa et al. reported that the surface charge of hydrogels dominates protein deposition: the hydrogel was synthesized with different ratios of positive, neutral, and negative monomers, respectively, to evaluate the surface charge [24]. Though they regulated the surface charge of the hydrogel, they did not shed light on the surface charge of proteins. Considering the environment in which actual contact lenses are used, not only ionic contact lenses, but also proteins may have different surface charges depending on the pH of ocular surface. Therefore, we investigated surface charge changes in contact lens materials and proteins caused by pH changes in the ocular surface. According to our findings, not only do these surface charges affect protein deposition, but also the level of protein deposition is increased despite the weakened surface charge for the protein. This indicates that the strength of the surface charge of contact lens materials has a greater effect on protein deposition than protein charges themselves.

### 3.4. Hydroxyl Group Content of Contact Lens

**Figure 4:**
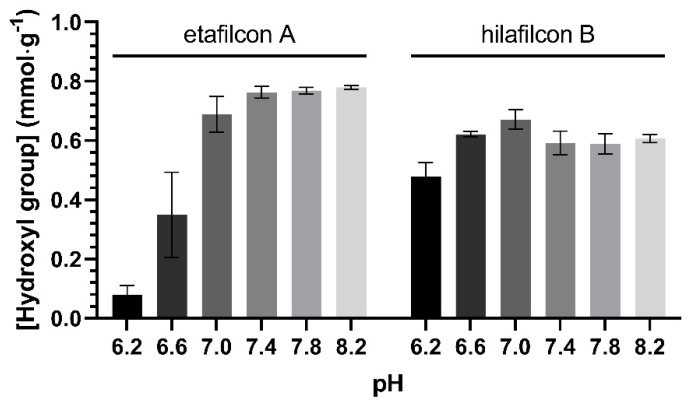
The hydroxyl group content in etafilcon A and hilafilcon B equilibrated in PBS solution (pH 6.2–8.2).

The hydroxyl group content of etafilcon A and hilafilcon B was measured to investigate their possible negative functional groups (Figure 4). The hydroxyl group content of etafilcon A and hilafilcon B ranged from 0.049 to 0.783 and 0.432 to 0.633 mmol∙g^−1^, respectively. As the pH increased, the hydroxyl group content also increased, implying that the MAA contained in etafilcon A can be expected to be ionized at basic pH conditions and fully ionized at 7.4.

The etafilcon A showed a statistically significant trend in the pH-dependency of the hydroxyl group content (Table 4), showing a relatively constant value above 7.4. This pH-dependent hydroxyl group content of etafilcon A strongly supports the hypothesis that the surface charge of etafilcon A was determined by the degree of ionization MAA.

The hilafilcon B showed a pH-dependent hydroxyl group content that reached statistical significance (Table 4). This tendency is expected to be owing to the hydroxyl group of poly(2-hydroxyethyl methacrylate)(HEMA). HEMA is a representative environmentally responsive biomaterial that responds to pH changes [33]. Though the content of hydroxyl groups changes in response to pH, it was probably insufficient to change the PZC of hilafilcon B.

## 4. Conclusions

In this study, we investigated protein deposition on contact lenses, using pH changes as the driving force of this phenomenon. The pH affected the surface charges of HEWL and BSA, as well as etafilcon A, but not hilafilcon B. Only etafilcon A and HEWL showed a pH-dependent tendency of protein deposition with statistical significance: increased protein deposition with increasing pH. Interestingly, the surface charge of HEWL became weaker with increasing pH, whereas that of etafilcon A became more negative. Therefore, it was found that the electrostatic interaction between the protein and contact lens was primarily governed by the surface charge of the contact lens.

The remarkably high level of HEWL deposition on etafilcon A has been widely observed. The presence of MAA in etafilcon A and its degree of ionization were the cause of high HEWL deposition. Here, we find clear evidence that not only the presence of MAA, but also its degree of ionization regulate the deposition of positive molecules.

## Figures and Tables

**Table 1:** Contact lens material types and classification.

Proprietary Name	1-Day ACUVUE^®^ MOIST	SofLens Daily Disposable
**Manufacturer**	Johnson & Johnson Vision Care	Bausch + Lomb Inc.
**FDA Group**	Ⅳ	Ⅱ
USAN *	etafilcon A	hilafilcon B
**Principal monomers ^†^**	HEMA–MAA + PVP	HEMA + NVP
**Water content (%)**	58	59

* United States adopted name. ^†^ HEMA, hydroxyethyl methacrylate; MAA, methacrylic acid; PVP, polyvinylpyrrolidone; NVP, N-Vinyl-2-pyrrolidone.

**Table 2:** The results of statistical analysis on the amount of deposited HWEL and BSA on etafilcon A and hilafilcon B depend on the pH. A repeated measures Kruskal–Wallis test was used for statistical analysis; * *p* < 0.05.

Contact Lens Material	Protein	pH	n	Mean Rank	χ^2^	*p*-Value
etafilcon A	HEWL	6.2	3	2.00	14.848	0.011 *
		6.6	3	5.00		
		7.0	3	10.67		
		7.4	3	17.00		
		7.8	3	10.33		
		8.2	3	12.00		
	BSA	6.2	3	2.67	6.474	0.263
		6.6	3	10.67		
		7.0	3	11.67		
		7.4	3	12.00		
		7.8	3	9.00		
		8.2	3	11.00		
hilafilcon B	HEWL	6.2	3	2.33	10.544	0.061
		6.6	3	9.33		
		7.0	3	7.33		
		7.4	3	13.67		
		7.8	3	14.67		
		8.2	3	9.67		
	BSA	6.2	3	7.00	7.550	0.183
		6.6	3	10.67		
		7.0	3	15.00		
		7.4	3	11.33		
		7.8	3	9.00		
		8.2	3	4.00		

**Table 3:** The results of statistical analysis on the PZC of etafilcon A and hilafilcon B equilibrated in PBS solution (pH 6.2-8.2). A repeated measures Kruskal–Wallis test was used for statistical analysis; * *p* < 0.05.

Contact Lens Material	pH	n	Mean Rank	χ^2^	*p*-Value
etafilcon A	6.2	3	2.33	12.415	0.030 *
	6.6	3	5.00		
	7.0	3	10.00		
	7.4	3	11.67		
	7.8	3	14.67		
	8.2	3	13.33		
hilafilcon B	6.2	3	5.00	3.297	0.654
	6.6	3	4.50		
	7.0	3	6.50		
	7.4	3	10.50		
	7.8	3	8.50		
	8.2	3	7.00		

**Table 4:** The results of statistical analysis on the hydroxyl group content of etafilcon A and hilafilcon B equilibrated in PBS solution (pH 6.2–8.2). A repeated measures Kruskal–Wallis test was used for statistical analysis; * *p* < 0.05.

Contact Lens Material	pH	n	Mean Rank	χ^2^	*p*-Value
etafilcon A	6.2	3	2.00	15.019	0.010 *
	6.6	3	5.00		
	7.0	3	8.33		
	7.4	3	12.67		
	7.8	3	13.00		
	8.2	3	16.00		
hilafilcon B	6.2	3	2.00	12.798	0.025 *
	6.6	3	12.00		
	7.0	3	17.00		
	7.4	3	8.17		
	7.8	3	8.67		
	8.2	3	9.17		

## Data Availability

The datasets generated and analyzed during the current study are available from the corresponding author on reasonable request.

## References

[1] Garrett Q., Chatelier R.C., Griesser H.J., Milthorpe B.K. (1998). Effect of charged groups on the adsorption and penetration of proteins onto and into carboxymethylated poly (HEMA) hydrogels. Biomaterials.

[2] Nicolson P.C., Vogt J. (2001). Soft contact lens polymers: An evolution. Biomaterials.

[3] Castillo E.J., Koenig J.L., Anderson J.M. (1986). Characterization of protein adsorption on soft contact lenses: IV. Comparison of in vivo spoilage with the in vitro adsorption of tear proteins. Biomaterials.

[4] Gachon A.M., Bilbault T., Dastugue B. (1986). Protein migration through hydrogels: A tool for measuring porosity—Application to hydrogels used as contact lenses. Anal. Biochem..

[5] Garrett Q., Garrett R.W., Milthorpe B.K. (1999). Lysozyme sorption in hydrogel contact lenses. Investig. Ophthalmol. Vis. Sci..

[6] Garrett Q., Laycock B., Garrett R.W. (2000). Hydrogel lens monomer constituents modulate protein sorption. Investig. Ophthalmol. Vis. Sci..

[7] Soltys-Robitaille C.E., Ammon D.M., Valint P.L., Grobe G.L. (2001). The relationship between contact lens surface charge and in-vitro protein deposition levels. Biomaterials.

[8] Lord M.S., Stenzel M.H., Simmons A., Milthorpe B.K. (2006). The effect of charged groups on protein interactions with poly (HEMA) hydrogels. Biomaterials.

[9] Green-Church K.B., Nichols J.J. (2008). Mass spectrometry-based proteomic analyses of contact lens deposition. Mol. Vis..

[10] Kramann C., Boehm N., Lorenz K., Wehrwein N., Stoffelns B.M., Pfeiffer N., Grus F.H. (2011). Effect of contact lenses on the protein composition in tear film: A ProteinChip study. Graefes Arch. Clin. Exp. Ophthalmol..

[11] Omali N.B., Zhao Z., Zhu H., Tilia D., Willcox M.D. (2013). Quantification of individual proteins in silicone hydrogel contact lens deposits. Mol. Vis..

[12] Omali N.B., Subbaraman L.N., Coles-Brennan C., Fadli Z., Jones L.W. (2015). Biological and clinical implications of lysozyme deposition on soft contact lenses. Optom. Vis. Sci..

[13] Deng X., Korogiannaki M., Rastegari B., Zhang J., Chen M., Fu Q., Sheardown H., Filipe C.D., Hoare T. (2016). ‘Click’ chemistry-tethered hyaluronic acid-based contact lens coatings improve lens wettability and lower protein adsorption. ACS Appl. Mater. Interfaces.

[14] Rabiah N.I., Scales C.W., Fuller G.G. (2019). The influence of protein deposition on contact lens tear film stability. Colloids Surf. B Biointerfaces.

[15] Sillero A., Ribeiro J.M. (1989). Isoelectric points of proteins: Theoretical determination. Anal. Biochem..

[16] Babić M.M., Antić K.M., Vuković J.S.J., Božić B.Đ., Davidović S.Z., Filipović J.M., Tomić S.L. (2015). Oxaprozin/poly (2-hydroxyethyl acrylate/itaconic acid) hydrogels: Morphological, thermal, swelling, drug release and antibacterial properties. J. Mater. Sci..

[17] Gulicovski J.J., Čerović L.S., Milonjić S.K. (2008). Point of zero charge and isoelectric point of alumina. Mater. Manuf. Process..

[18] Taddei P., Balducci F., Simoni R., Monti P. (2005). Raman, IR and Thermal Study of a New Highly Biocompatible Phosphorylcholine-Based Contact Lens. J. Mol. Struct..

[19] Serjeant E.P., Dempsey B. (1979). Ionisation Constants of Organic Acids in Aqueous Solution.

[20] Efron N., Maldonado-Codina C. (2017). Comprehensive Biomaterials.

[21] Norn M.S. (1988). Tear Fluid pH in Normals, Contact Lens Wearers, and Pathological Cases. Acta. Ophthalmol..

[22] Coles W.H., Jaros P.A. (1984). Dynamics of Ocular Surface pH. Brit. J. Ophthalmol..

[23] Chatterjee S., Upadhyay P., Mishra M., Srividya M., Akshara M.R., Kamali N., Zaidi Z.S., Iqbal S.F., Misra S.K. (2020). Advances in Chemistry and Composition of Soft Materials for Drug Releasing Contact Lenses. Rsc. Adv..

[24] Guo H., Uehara Y., Matsuda T., Kiyama R., Li L., Ahmed J., Katsuyama Y., Nonoyama T., Kurokawa T. (2020). Surface Charge Dominated Protein Absorption on Hydrogels. Soft Matter.

[25] Ahn J., Choi M. (2021). Binding Affinity of Benzalkonium Chloride on Contact Lens Surfaces and the Effects on Their Physical Properties. Colloids Surf. B Biointerfaces.

[26] Shin S., Choi M. (2017). Equilibrium Study of Copper Absorption to Different Types of Soft Contact Lens. Appl. Biol. Chem..

[27] Zhou Y., Zhang M., Hu X., Wang X., Niu J., Ma T. (2013). Adsorption of Cationic Dyes on a Cellulose-Based Multicarboxyl Adsorbent. J. Chem. Eng. Data..

[28] Yuan Z., Wang J., Wang Y., Liu Q., Zhong Y., Wang Y., Li L., Lincoln S.F., Guo X. (2019). Preparation of a Poly(Acrylic Acid) Based Hydrogel with Fast Adsorption Rate and High Adsorption Capacity for the Removal of Cationic Dyes. Rsc. Adv..

[29] Musgrave C.S.A., Fang F. (2019). Contact Lens Materials: A Materials Science Perspective. Materials.

[30] Alderton G., Ward W.H., Fevold H.L. (1945). Isolation of lysozyme from egg white. J. Biol. Chem..

[31] Trivedi V.D., Raman B., Rao C.M., Ramakrishna T. (1997). Co-refolding denatured-reduced hen egg white lysozyme with acidic and basic proteins. FEBS Lett..

[32] Rezwan K., Meier L.P., Rezwan M., Vörös J., Textor M., Gauckler L.J. (2004). Bovine serum albumin adsorption onto colloidal Al2O3 particles: A new model based on zeta potential and UV-vis measurements. Langmuir.

[33] Sari S.M.C., Benmouna M., Mahlous M., Kaci M. (2013). Swelling behavior of poly (2-hydroxyethyl methacrylate) copolymer gels. MATEC Web Conf..

